# One-pot synthesis of CdS/metal–organic framework aerogel composites for efficient visible photocatalytic reduction of aqueous Cr(vi)†

**DOI:** 10.1039/c9ra08339a

**Published:** 2019-11-19

**Authors:** Haiyan Yang, Liang Jiang, Wei Wang, Zhifang Luo, Jing Li, Zijuan He, Zhiyin Yan, Jiaqiang Wang

**Affiliations:** National Center for International Research on Photoelectric and Energy Materials, Yunnan Provincial Collaborative Innovation Center of Green Chemistry for Lignite Energy, Yunnan Province Engineering Research Center of Photocatalytic Treatment of Industrial Wastewater, School of Chemical Sciences & Technology, Yunnan University Kunming 650091 China jqwang@ynu.edu.cn; School of Energy, Yunnan University Kunming 650091 China

## Abstract

Metal–organic framework aerogels (MOAs) embedded with CdS (CdS/MOA(Cr)) synthesized *via* a facile one-pot solvothermal method have a larger surface area than pristine MOA(Cr) and the post-synthesized composite. CdS/MOA(Cr) exhibited 5 times enhancement in the photocatalytic activity than that of pure CdS for Cr(vi) reduction under visible light without adding any sacrificial agent, due to the larger surface area and photosensitazation of MOA(Cr).

## Abstract 2

Metal–organic framework aerogels (MOAs) embedded with CdS (CdS/MOA(Cr)) synthesized *via* a facile one-pot solvothermal method have a larger surface area than pristine MOA(Cr) and the post-synthesized composite.

## Graphical abstract

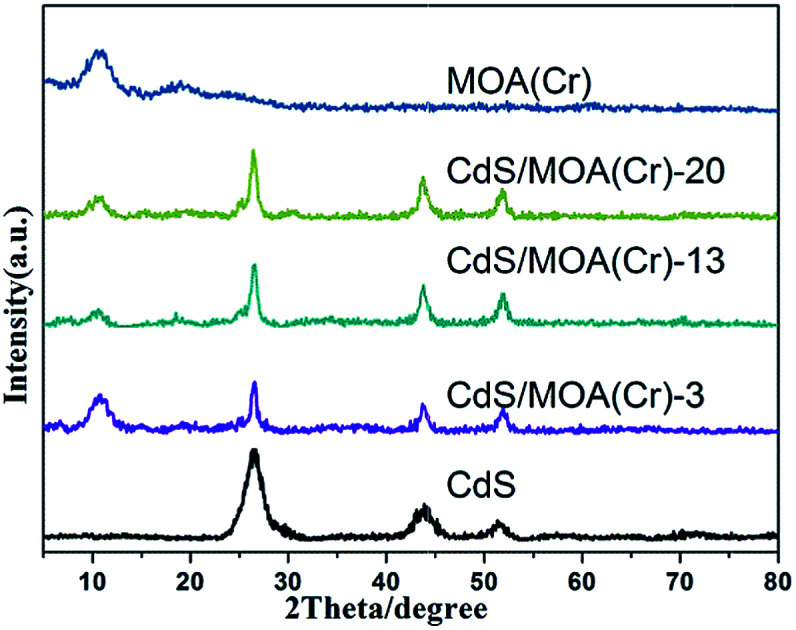

Cr(vi) is a common contaminant in water, and is a potentially carcinogenic contaminant.^1^ In recent years, photocatalytic reduction has been widely regarded as a promising method to remove Cr(vi).^2^ A number of systems such as those doped with transition metal ions or nonmetallic ions,^3^ or coupled with semiconductors,^4^ have been reported to serve as candidates for this application.

Hybrid CdS-based nanostructures, as a significant class of multicomponent heterogeneous architectures, could bring unexpected properties for improving the potential applications of CdS.^16^ For instance, metal sulphide nanomaterials such as ZnIn_2_S_4_ nanosheet-coated CdS nanorod heterostructures, CuFe_2_O_4_/CdS, CdS QD-Decorated Self-Doped γ-Bi_2_MoO_6_ and CdS–Sn_2_S_3_ dispersed onto reduced graphene oxide are widely used for the photocatalytic reduction of Cr(vi).^4,5^

On the other hand, metal–organic frameworks (MOFs) have aroused widespread interest due to their porous crystalline frameworks, high surface areas and the feature of light harvesting.^6^ Recent researches have demonstrated the possibility of using MOFs in photocatalytic reduction of aqueous Cr(vi). For example, MIL-53(Fe) exhibited photocatalytic activity for the reduction of Cr(vi) in the mixed system(Cr(vi)/dyes).^7^ NH_2_-UiO-66 showed high photocatalytic activity for the reduction of Cr(vi) in the methanol/Cr(vi) system at pH 2.^8^

However, these MOFs are not as effective as that of metal sulphides. We found that embedding of CdS on MOFs could significantly increase the photocatalytic efficiency of CdS for visible light-driven hydrogen production.^9^ Recently, CdS, In_2_S_3_, SnS_2_, Sb_2_S_3_ quantum dots (QDs) into MIL-125(Ti) have shown the photocatalytic activity for reduction of Cr(vi), due to the photosensitizing effect and the enhanced light harvesting efficiency.^10^ Nevertheless, all these hybrid metal sulphides and MOFs nanostructures were prepared *via* post-synthesis method. Therefore, these composites would result in the decrease of the surface area compared with pristine MOF.

To improve the physical and chemical properties of MOFs, metal–organic framework aerogels (MOAs) have emerged as a new porous material. MOAs are prepared from metal–organic gels and the liquid in the gels has been replaced with gas by supercritical drying, which achieve high porosity values as MOFs, low density and interconnected networks structure.^11^ MOAs are amorphous and interconnected coherent network structure which is significantly different from MOFs.^12^ Thus, MOAs have exhibited some advantages over MOFs. For example, MOA(Cr) based on carboxyl porphyrins and MOA(Al) have a fairly better capability in dye adsorption than that of MOFs crystalline material.^13^ MOA(Fe) showed high efficacy in removal of arsenic in water.^14^ Therefore, it will be a good possibility to apply MOAs and their composites hybrided with CdS as efficient photocatalysts which has not been reported as far as we know.

Herein, we reported a simple and effective strategy to one-pot synthesis of CdS embedded in MOA(Cr) (CdS/MOA(Cr)) composites for the first time. Interestingly, we found that the photocatalytic activity could be significantly improved by embedding CdS in MOA(Cr) than in MIL-100(Cr) and CdS/MOA(Cr) prepared by post-synthesis method.

CdS/MOA(Cr) was synthesized by a facile one-pot solvothermal treatment. The weight ratios of CdS in samples were 3, 13 and 20%, respectively, which were labelled as CdS/MOA(Cr)-*X*, where *X* = 3, 13 and 20%. For comparison, CdS/MOA(Cr) was also prepared by post-synthesis method. MOA(Cr) was synthesized first and then 20% CdS was deposited on MOA(Cr) by solvothermal method, which was named as CdS/MOA(Cr)-2-post. X-ray diffraction (XRD) measurements (Fig. 1) proved that MOA(Cr) exhibited weak and broad peaks, which confirmed MOA(Cr) was successfully prepared. The embedded CdS into MOA(Cr) was also successfully synthesized and the three diffraction peaks were corresponded to cubic phase of CdS.^15^ The average crystallite sizes for the embedded CdS were between 7 to 9 nm which were calculated using the Scherrer formula for the (111) facet diffraction peak. The XRD pattern was different at the molecular level from MIL-100(Cr) (Fig. S1†).^12*b*^

**Fig. 1:**
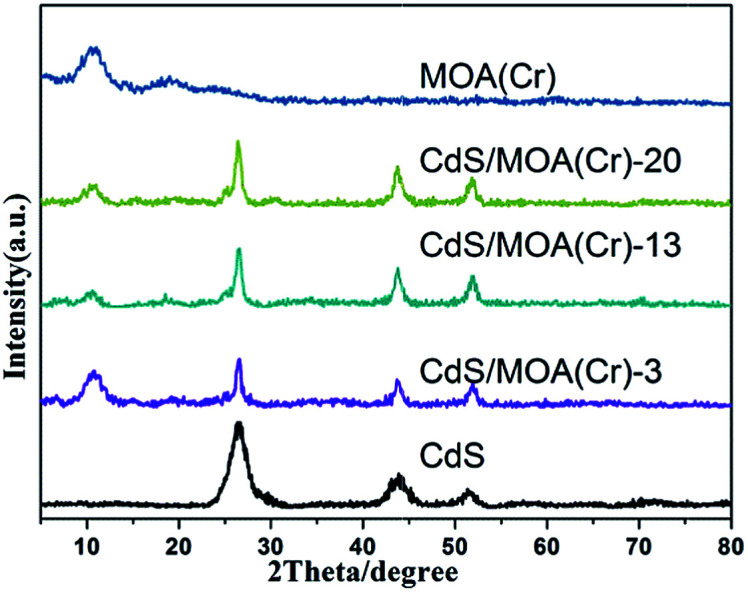
XRD patterns of the samples.

The N_2_ adsorption–desorption isotherm (Fig. S2†) of MOA(Cr) and CdS/MOA(Cr) exhibited typical isotherm of type I having inflection around *P*/*P*_0_ = 0.4–0.8 and type II isotherm was observed on pure CdS. The Brunauer–Emmett–Teller (BET) surface areas, pore volumes and pore sizes of samples are listed in Table 1. The Brunauer–Emmett–Teller (BET) surface area (*S*_BET_) of MOA(Cr) is 629.2 m^2^ g^−1^, which also confirms that MOA(Cr) was properly synthesized. Surprisingly, the *S*_BET_ of CdS/MOA(Cr) were much higher than that of MOA(Cr), mainly because the adding anions may affect the coordinative crosslinking of the samples.^16^ The pore volume and pore size were rarely changed after CdS incorporation in MOA(Cr). While the *S*_BET_ and pore values of CdS/MOA(Cr)-2-post synthesized *via* post-synthesis methods were much lower than that of MOA(Cr). In general, the loaded CdS in MOA(Cr) by post-synthesis method may block pores of MOA(Cr). Similarly, the *S*_BET_ and pore volume also decrease when CdS loading in MIL-100(Cr). This is attributed to pore blockage by CdS nanoparticles.^9^

**Table 1:** Summary of textural properties of the samples

Samples	*S*_BET_ (m^2^ g^−1^)	Pore volume (cm^3^ g^−1^)	Pore size (nm)
MOA(Cr)	629.2	0.4	3.3
CdS/MOA(Cr)-3	821.6	0.6	3.1
CdS/MOA(Cr)-13	829.8	0.6	3.2
CdS/MOA(Cr)-20	714.4	0.5	3.1
MIL-100	1518.8	0.9	2.4
CdS/MIL-100-20	1202.9	0.7	2.3
CdS/MOA(Cr)-2-post	312.9	0.2	2.3
CdS	131.8	0.1	5.9

The scanning electron microscopy (SEM) images present spongy porous structures with spherical interconnected particles (Fig. 2). CdS/MOA(Cr)-20 show more spongy porous structure than pure MOA(Cr), which is consistent with that observed from the BET surface areas. In Fig. 2(c), the deposition of CdS is irregular spherical aggregate covered on the surface of MOA(Cr). The transmission electron microscope (TEM) of CdS/MOA(Cr)-20 reveals the fluffy and irregular aggregates (Fig. 2). High resolution TEM reveals a lattice spacing of 0.34 nm corresponding to the interplanar distance between adjacent (111) crystallographic planes of cubic CdS. Fourier transform infrared (FT-IR) spectra of CdS/MOA(Cr)-20 and CdS/MIL-100-20 are shown in Fig. 3(a). Strong absorptions at *ca.* 1620 cm^−1^ correspond to C=C stretching vibrations in phenyl ring and the band observed at about 1727 cm^−1^ is related to C=O stretching vibrations.^17^ The bands observed at *ca.* 3430 cm^−1^ correspond to O–H stretching of water molecules. The FT-IR spectra show that coordination bonds are formed between Cr^3+^ ions and carboxylate groups.^17*b*,18^

**Fig. 2:**
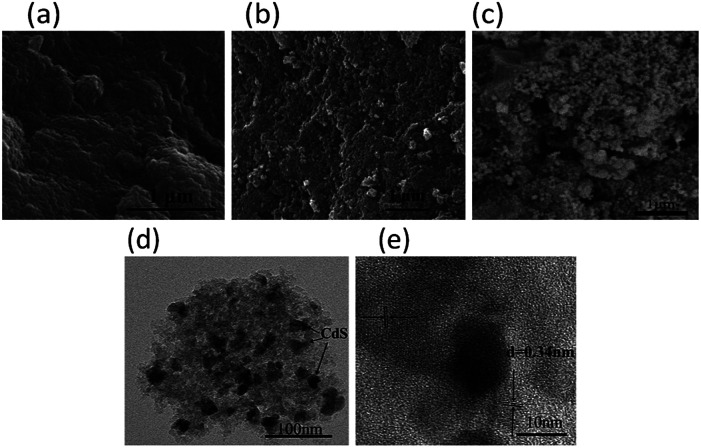
SEM image of (a) MOA(Cr), (b) CdS/MOA(Cr)-20 and (c) CdS/MOA(Cr)-2-post. (d) TEM image and (e) HRTEM image of CdS/MOA(Cr)-20.

**Fig. 3:**
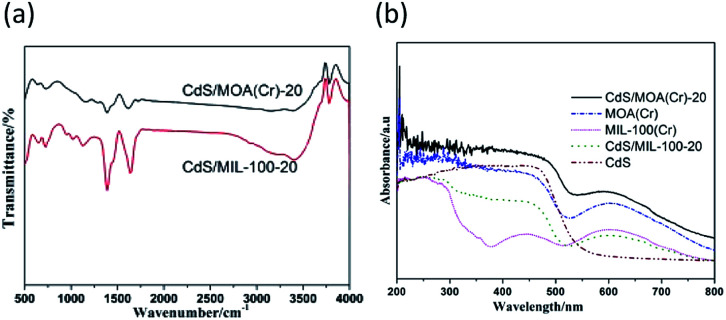
(a) Fourier transform infrared spectra and (b) UV-vis diffuse reflectance spectra of different photocatalysts.

Fig. 3(b) and S3(a)† shows the UV-vis diffuse reflectance spectra of the samples. The absorption-band of MOA(Cr) in the UV region can be assigned to the π–π* transition of ligands, whereas the absorption-band in the visible region ascribe to the d–d spin-allowed transition of the Cr^3+^.^9,19^ MOA(Cr) revealed significant higher absorption in the visible range than that of MIL-100(Cr). It can be attributed to the low temperature of the as-synthesized MOA(Cr).^11*d*^ CdS/MOA(Cr) displays mixed absorption feature of pure CdS and MOA(Cr), which was consistent with the colour change from deep green to deep yellow-green. Furthermore, the absorption edges of CdS/MOA(Cr) showed clear red-shift at the visible light range. The band gaps of the CdS, MOA(Cr) and CdS/MOA(Cr)-20 were calculated from Tauc plot (Fig. S3(b)†). The band gaps of CdS, MOA(Cr) and CdS/MOA(Cr)-20 are estimated as 2.1, 2.0 and 1.7 eV, respectively. The result suggests the potential photocatalytic activity of CdS/MOA(Cr) under visible-light irradiation.

The photoelectron chemical behaviors of CdS/MOA(Cr)-20 and CdS/MIL-100-20 were investigated (Fig. S4†). The results showed that CdS/MOA(Cr)-20 possessed a superior photocurrent transient response compared with its MOFs counterpart. Then, the samples were evaluated in photocatalytic reduction of Cr(vi) without any adding sacrificial agent in a quartz reactor under visible light irradiation. The photocatalytic activities results are presented in Fig. 4 and S5.† It is observed that no meaningful activity for photocatalytic reduction of Cr(vi) was detected in the presence of pure MOA(Cr) or in the absence of any photocatalyst under visible light irradiation. By contrast, the photocatalytic reduction yields of Cr(vi) improved with the increase of irradiation time in the presence of CdS/MOA(Cr), MIL-100/CdS and pure CdS. This indicates that the photocatalytic reduction of Cr(vi) did not occur by MOA(Cr) alone. The photocatalytic yield of Cr(vi) of pure CdS was 20%. When CdS was introduced into MOA(Cr), the photocatalytic yield was increased notably. After embedding only 3 wt% of CdS into MOA(Cr), the photocatalytic yield of Cr(vi) was increased to 59%. The maximum photocatalytic yield of Cr(vi) was found at 20 wt% CdS (*ca.* 99%), that was higher than that of CdS/MIL-100-20 (*ca.* 74%). Furthermore, the XRD patterns, Cr 2p and O 1s spectra of fresh CdS/MOA(Cr)-20 and after used CdS/MOA(Cr)-20 were displayed in Fig. S5(b)–(d),† respectively. The XRD peaks did not shift after used CdS/MOA(Cr)-20. The binding energy of Cr and O were almost unchanged. The results prove no significant change in CdS/MOA(Cr)-20 after the photocatalytic reaction process.

**Fig. 4:**
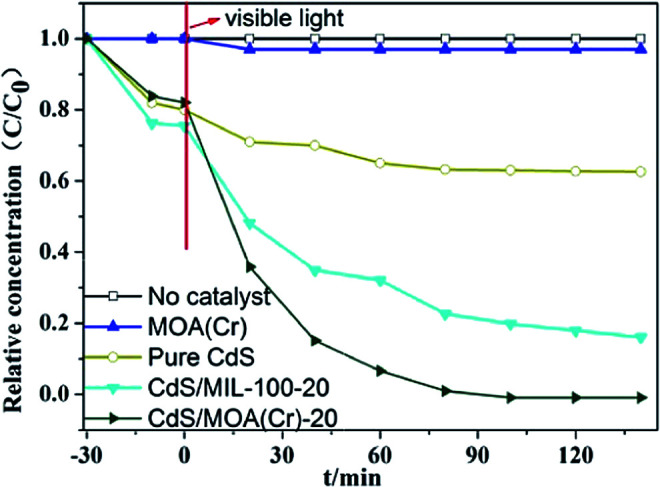
Photocatalytic activities of photocatalysts for the reduction of Cr(vi) under visible light.

The photocatalytic reduction rate constant (*k*) of Cr(vi) over different samples were calculated by the pseudo-first-order model. The linear plots of ln(*C*_o_/*C*) *vs.* irradiation time was observed in Fig. S6.† The correlation coefficient *R*^2^ and corresponding values of *k* were provided in Table 2. The rate constants of photocatalytic reduction for Cr(vi) was calculated to be 5.3 × 10^−2^ min^−1^ for CdS/MOA(Cr)-20, which was approximately 8.8, 4.8, 3.8 and 1.5 times higher than that of CdS/MOA(Cr)-3, CdS/MOA(Cr)-13, CdS/MIL-100-20 and CdS/MOA(Cr)-2-step, respectively. This could be ascribed to the superior light absorption in the visible range of MOA(Cr). This highlights the significance of MOA(Cr) based material.

**Table 2:** Summary of rate constants of photocatalytic reduction of Cr(vi) over the samples

Samples	Rate contants (min^−1^)	*R* ^2^
CdS/MOA(Cr)-3	0.6 × 10^−2^	0.93
CdS/MOA(Cr)-13	1.1 × 10^−2^	0.98
CdS/MOA(Cr)-20	5.3 × 10^−2^	0.95
CdS/MIL-100-20	1.4 × 10^−2^	0.93
CdS/MOA(Cr)-2-post	3.6 × 10^−2^	0.98

The success in enhancement of the photocatalytic reduction activity of Cr(vi) by using CdS/MOA(Cr) encouraged us to extend this approach to other MOA. MOA(Al) was selected as the model MOA since it is the most-studied MOA. However, under same experimental conditions, CdS modified MOA(Al) exhibited very low activity (*ca.* 10%) for photocatalytic reduction of Cr(vi) (Fig. S5†). This is because that CdS/MOA(Al)-20 did not exhibit any significant absorption in the visible range. The Fig. S4† shows the photocurrent transient response of CdS/MOA(Al)-20. Under visible light irradiation, the photocurrent transient response of CdS/MOA(Al)-20 is much lower than that of CdS/MOA(Cr)-20.

In addition, the valence bands (VBs) of CdS, MOA(Cr) and CdS/MOA(Cr)-20 were investigated by XPS. The measured XPS VB values of CdS, MOA(Cr) and CdS/MOA(Cr)-20 are 1.71, 1.32 and 1.24 eV, respectively (Fig. S7†). Correspondingly, the conduction bands (CBs) of CdS, MOA(Cr) and CdS/MOA(Cr)-20 are calculated to be −0.39, −0.68 and −0.46 eV, according to their values of band gap energy.

On the basis of the above discussion, a probable mechanism for the photocatalytic reduction is proposed: irradiated light was absorbed mainly by MOA(Cr) photosensitizer unit and partly by CdS. Under visible light, an excited state of MOA(Cr)* is formed and electrons are transferred from the excited states of MOA(Cr) to the conduction band of CdS. Furthermore, CdS could also be excited by absorbing photons of visible light. The conduction band of CdS is more negative than Cr(vi)/Cr(iii) potential (0.51 V *vs.* NHE).^7,20^ Then the photo-induced electrons accumulated on CdS can reduce Cr(vi) to Cr(iii). Meanwhile, water molecules can be oxidized by the holes and the excited state MOA(Cr) species return to the ground state.^21^ MOA(Cr) improves the photoinduced electron–hole charge separation and weak the possibility of charge recombination, as well as an ideal host for CdS nanoparticles, which makes it a good photocatalytic reduction activity of Cr(vi). Additionally, the enhancement of the photocatalytic activity of CdS embedded on MOA(Cr) can also be attributed to their high surface area and produce more active sites which enhanced photocatalytic activity.

The photocatalytic activity of CdS/MOA(Cr) prepared by post-synthesis method was lower than that of CdS/MOA(Cr) prepared by one-pot method. This is due to the CdS nanoparticles just deposited on the surface of MOA(Cr) but not embedded into MOA(Cr) after post-system method (Fig. S8†) and the specific surface area was decreased. Therefore, when CdS/MOA(Cr) prepared by one-pot method as catalyst, the interconnected network structures of could adsorb more light energy onto the catalyst, the aerogel molecules might promote the spread of light and electron transport.^11*d*,12*b*^

In summary, we have demonstrated for the first time that one-pot synthesis of CdS/MOA(Cr) composites significantly increases the photocatalytic reduction activity of Cr(vi). Through one-pot method, the CdS nanoparticles were embedded into MOA(Cr) and the surface area were increased. The experimental results indicate that CdS/MOA(Cr) composites exhibit a better photocatalytic performance than pure CdS and the photocatalytic performance of CdS/MOA(Cr) is dependent on the MOA(Cr) photosensitizer unit which could increase light absorption intensity and charge separation.

## Conflicts of interest

There are no conflicts to declare.

## Supplementary Material

RA-009-C9RA08339A-s001

## References

[cit1] Marinho B. A., Cristóvão R. O., Djellabi R., Loureiro J. M., Boaventura R. A. R., Vilar V. J. P. (2017). Appl. Catal., B.

[cit2] Wang C.-C., Du X.-D., Li J., Guo X.-X., Wang P., Zhang J. (2016). Appl. Catal., B.

[cit3] Wang Y., Yang W., Zhang L., Hu Y., Lou X. W. (2013). Nanoscale.

[cit4] Xue C., Yan X., An H., Li H., Wei J., Yang G. (2018). Appl. Catal., B.

[cit5] Kandi D., Martha S., Thirumurugan A., Parida K. M. (2017). ACS Omega.

[cit6] Liu X., Dang R., Dong W., Huang X., Tang J., Gao H., Wang G. (2017). Appl. Catal., B.

[cit7] Liang R., Jing F., Shen L., Qin N., Wu L. (2015). J. Hazard. Mater..

[cit8] Shen L., Liang S., Wu W., Liang R., Wu L. (2013). Dalton Trans..

[cit9] He J., Yan Z., Wang J., Xie J., Jiang L., Shi Y., Yuan F., Yu F., Sun Y. (2013). Chem. Commun..

[cit10] Wang H., Yuan X., Wu Y., Chen X., Leng L., Zeng G. (2015). RSC Adv..

[cit11] Saraji M., Shahvar A. (2017). Anal. Chim. Acta.

[cit12] Zhang J., Su C.-Y. (2013). Coord. Chem. Rev..

[cit13] Zhao X., Yuan L., Zhang Z.-q., Wang Y.-s., Yu Q., Li J. (2016). Inorg. Chem..

[cit14] Gao Z., Sui J., Xie X., Li X., Song S., Zhang H., Hu Y., Hong Y., Wang X., Cui J., Hao J. (2018). AIChE J..

[cit15] Yang X., Yan Z., Jiang L., Wang X., Zheng K., Wang Y., Li Q., Wang J. (2013). Procedia Environ. Sci..

[cit16] Wei S.-C., Pan M., Li K., Wang S., Zhang J., Su C.-Y. (2014). Adv. Mater..

[cit17] Hu Y., Fan Y., Huang Z., Song C., Li G. (2012). Chem. Commun..

[cit18] Chen J. H., Xing H. T., Guo H. X., Li G. P., Weng W., Hu S. R. (2013). J. Hazard. Mater..

[cit19] He J., Yang H., Chen Y., Yan Z., Zeng Y., Luo Z., Gao W., Wang J. (2015). Water, Air, Soil Pollut..

[cit20] Liu X., Pan L., Lv T., Zhu G., Sun Z., Sun C. (2011). Chem. Commun..

[cit21] Qiu J., Zhang X. F., Zhang X., Feng Y., Li Y., Yang L., Lu H., Yao J. (2018). J. Hazard. Mater..

