# Supplementary material for: One-pot synthesis of CdS/metal–organic framework aerogel composites for efficient visible photocatalytic reduction of aqueous Cr(vi)
Source: RSC Adv. 2019 Nov 19;9(64):37594–7. doi: 10.1039/c9ra08339a (PMC9075721; doi:10.1039/c9ra08339a)
Supplement: RA-009-C9RA08339A-s001 [file RA-009-C9RA08339A-s001.pdf]

Electronic Supplementary Material (ESI) for RSC Advances.
This journal is © The Royal Society of Chemistry 2019

Supporting information

One-pot synthesis of CdS/metal-organic framework aerogel composites for efficient visible photocatalytic reduction of aqueous Cr(VI)

Haiyan Yang,[a] Liang Jiang,[a] Wei Wang,[a] Zhifang Luo,[a] Jing Li,[b] Zijuan He,[a] Zhiyin Yan,[a] Jiaqiang Wang*[a]

[a] National Center for International Research on Photoelectric and Energy Materials, Yunnan Provincial Collaborative Innovation Center of Green Chemistry for Lignite Energy, Yunnan Province Engineering Research Center of Photocatalytic Treatment of Industrial Wastewater, School of Chemical Sciences & Technology, Yunnan University, Kunming 650091, China.

[b] School of Engergy, Yunnan University, Kunming 650091, China

* Correspondence: jqwang@ynu.edu.cn; Tel.: +86-871-6503-1567

Synthesis

MOA(Cr): 1,3,5-benzene tricarboxylic acid(H_3BTC,5mmol) and $Cr(NO_3)_3{\cdot}9H_2O$ (5mmol) and ethanol (30ml) were mixed in a Teflon liner and kept at 100°C for 10h. After gelation, the green wet gel was exchanged with ethanol for 1 d. The gel was transferred into a standard autoclave under nitrogen atmosphere and kept at 250°C for 1h. Then the pressure was quickly released by venting of solvent vapour. The sample was flushed again with nitrogen for 15min and allowed to cool down in nitrogen to room temperature.

MOA(Al): 1,3,5-benzene tricarboxylic acid(H_3BTC,5mmol) and $Al(NO_3)_3{\cdot}9H_2O$ (5mmol) and ethanol (30ml) were mixed in a Teflon liner and kept at 100°C for 10h. After gelation, the white wet gel was exchanged with ethanol for 1 d. The gel was transferred into a standard autoclave under nitrogen atmosphere and kept at 250°C for 1h. Then the pressure was quickly released by venting of solvent vapour. The sample was flushed again with nitrogen for 15min and allowed to cool down in nitrogen to room temperature.

CdS-embedded MOA: 1,3,5-benzene tricarboxylic acid(H_3BTC,5mmol) and $Cr(NO_3)_3{\cdot}9H_2O$ (5mmol) and ethanol (30ml) were mixed in a Teflon liner. After the addition of cadmium acetate dehydrate ($Cd(Ac)_2{\cdot}2H_2O$) and dimethyl sulfoxide (DMSO), the mixing solution kept at 100°C for 10h. After gelation, the green wet gel was exchanged with ethanol for 1 d. The gel was transferred into a standard autoclave

under nitrogen atmosphere and kept at 250°C for 1h. Then the pressure was quickly released by venting of solvent vapour. The sample was flushed again with nitrogen for 15min and allowed to cool down in nitrogen to room temperature. MOA(Cr) loaded with 3, 13, and 20% of CdS in weight was labelled as CdS/MOA(Cr)-X, X=3, 13, and 20%. CdS/MOA(Al)-20 was prepared using the same method and the $Cr(NO_3)_3{\cdot}9H_2O$ is substituted with $Al(NO_3)_3{\cdot}9H_2O$.

Pure CdS: Pure CdS was prepared using the same method in the absence of MOA(Cr).

MIL-100: A typical procedure reported by Férey[1] was used to synthesize MIL-100. Metallic chromium (260mg) was dispersed into hydrofluoric acid (1 mL) and distilled water (3 mL). After the addition of 1,3,5-benzene tricarboxylic acid (H_3BTC, 600 mg) and distilled water (24 mL), the mixing solution was heated in a Teflon-lined stainless autoclave , kept at 220 °C for 96 h, and then cooled to room temperature. The solid product was filtrated out and purified in dimethylformamide (DMF) overnight. The resulting green powder was successively washed with distilled water and acetone and finally dried in air at 90 °C.

CdS-embedded MIL-100: MIL-100 loaded with 20% CdS was carried out via a solvothermal process according to our previous report.[2]

Characterizations

X-ray powder diffraction (XRD) experiments were conducted on a Rigaku TTRAX III spectrometer with Cu Kα radiation. The data were recorded in the 2θ range 10°-90° at a scan rate of 10°/min. The Brunauer-Emmett-Teller (BET) surface areas were measured by the nitrogen adsorption/desorption measurements using a Micromeritics Tristar II Surface Area and Porosity Analyzer. Before the test, the samples were degassed in vacuum at 90 °C for 4 h. Scanning electron microscopy (SEM) were taken on FEI Quanta 200 ESEM scanning electron microscope operated at an accelerating voltage of 10 kV. High-resolution transmission electron microscopy (HRTEM) imagines were carried out on a JEM-2100 operated at 200 kV. UV-Vis diffuse reflectance spectra were measured on a Shimadzu UV-2401PC photometer over the range from 200 to 800 nm at room temperature. X-ray photoelectron spectroscopy (XPS) measurements were carried out on a PHI5500ESCA analyzer with 200 W Mg Kα radiation. The base pressure was about ~10−7 Pa and the binding energies were referenced to the C1s line at 284.8 eV from adventitious carbon. Fourier transform infrared (FT-IR) spectra were obtained on a Thermo Nicolet 8700 instrument.

Photocatalytic activity

The photocatalytic reduction were carried out in a quartz photoreactor containing 50 mL 10ppm Cr(VI) aqueous solution and 50 mg of photocatalyst. The suspensions were homogenized in the dark for 3 h to attain adsorption–desorption equilibrium. The suspensions were irradiated by 350W Xe lamp with a coloured glass filter (>420 nm), was utilized for the purpose of allowing only visible-light radiation. The solutions were stirred during the reaction process with a magnetic stirrer. During illumination, about 5 mL of suspension was filtered to separate the catalyst. The Cr(VI) content was determined at 540 nm using the 722 UV–vis spectrophotometer.

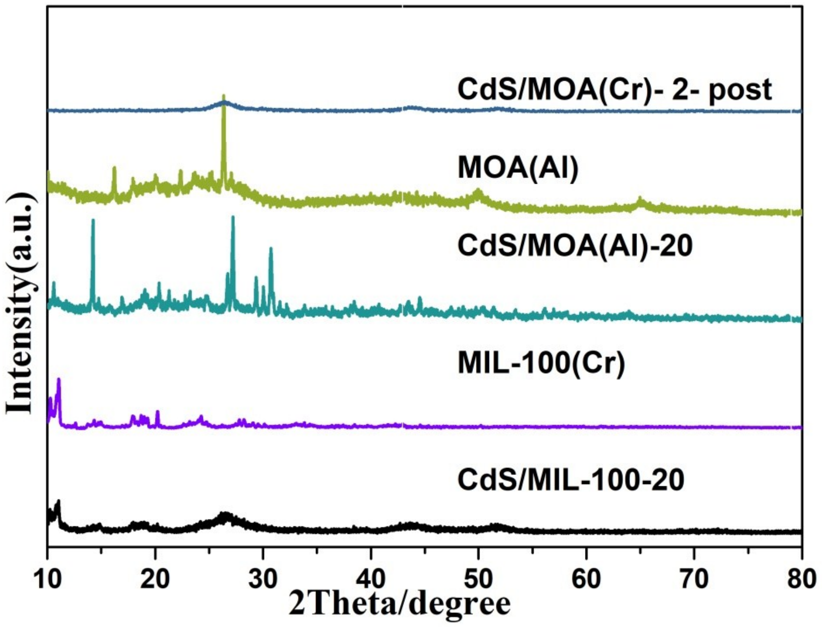

Fig.S1. XRD patterns of CdS/MOA(Cr)-2-post, MOA(Al), CdS/MOA(Al)-20, MIL-100(Cr)and CdS/MIL-100-20.

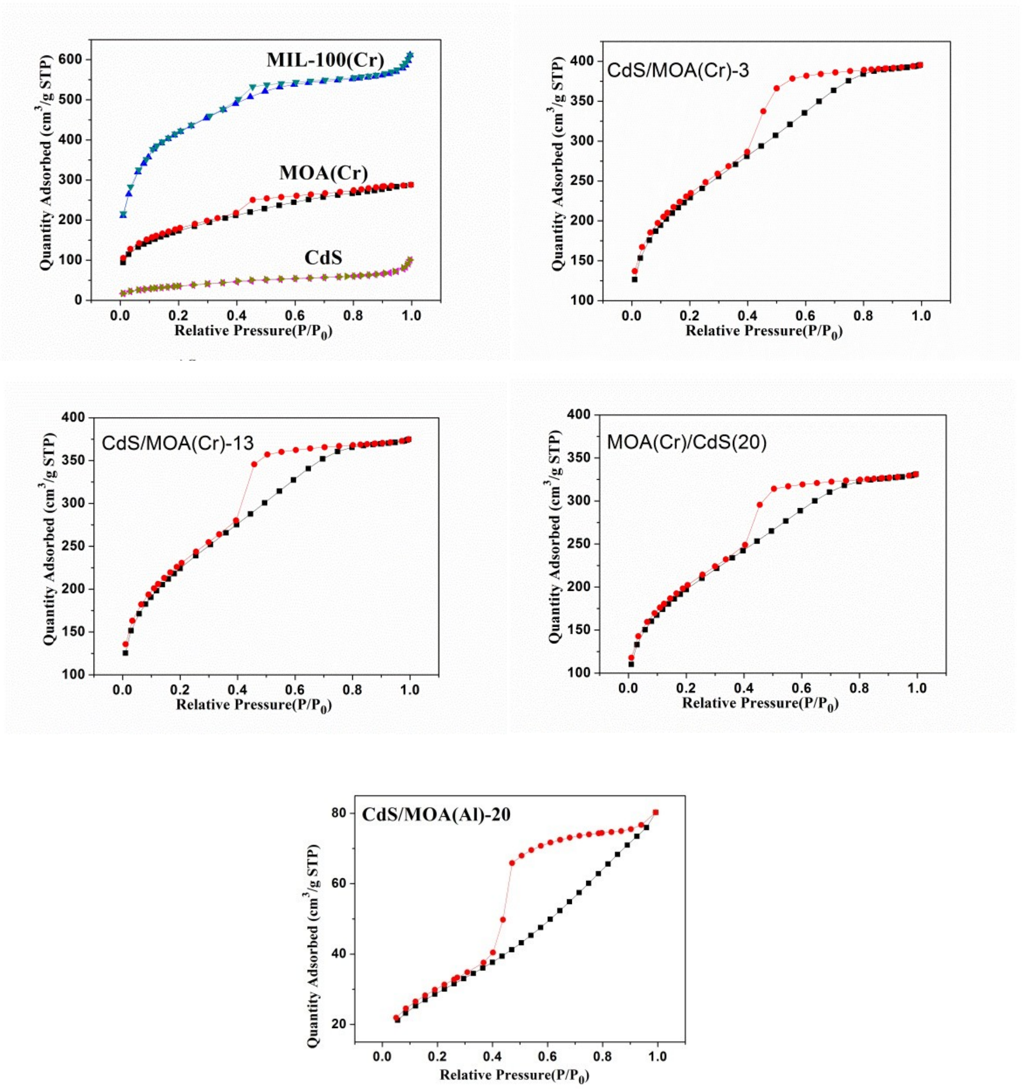

Fig.S2. N_2 adsorption/desorption isotherms of samples.

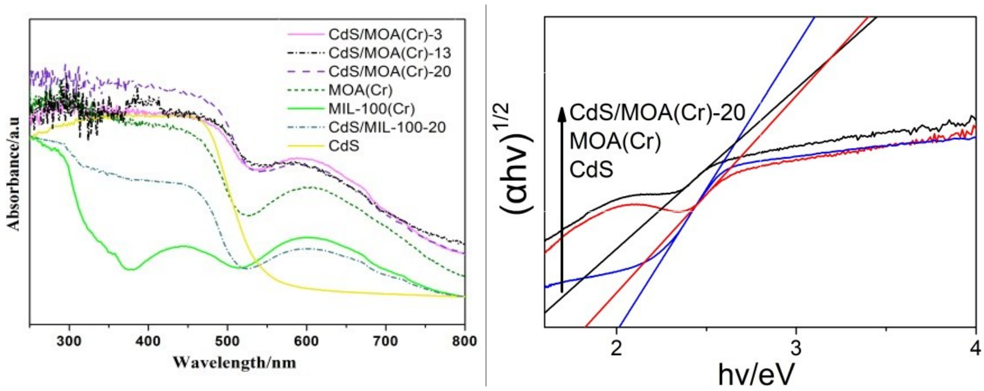

Figure S3. (a)UV-Vis diffuse reflectance spectra for different photocatalysts; (b) estimated band gap energy of CdS/MOA(Cr)-20, MOA(Cr) and CdS.

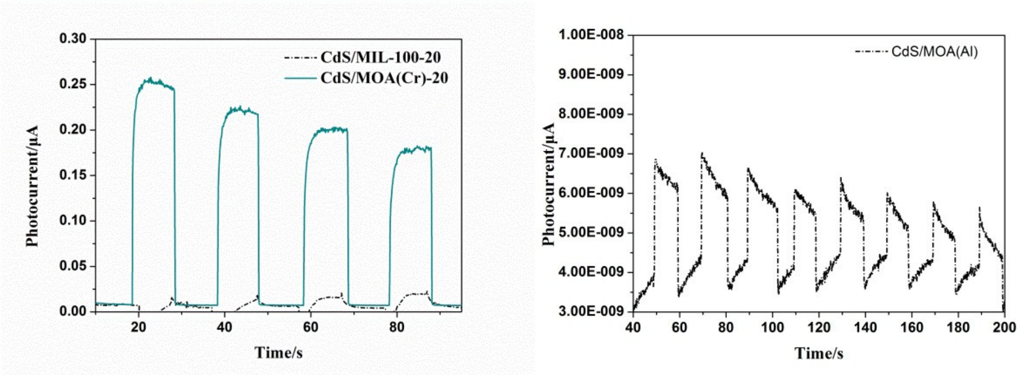

Fig.S4. Periodic on/off photocurrent output of CdS/MIL-100-20, CdS/MOA(Cr)-20 and CdS/MOA(Al) casted on ITO glass in a 0.2M Na_2SO_4 solution.

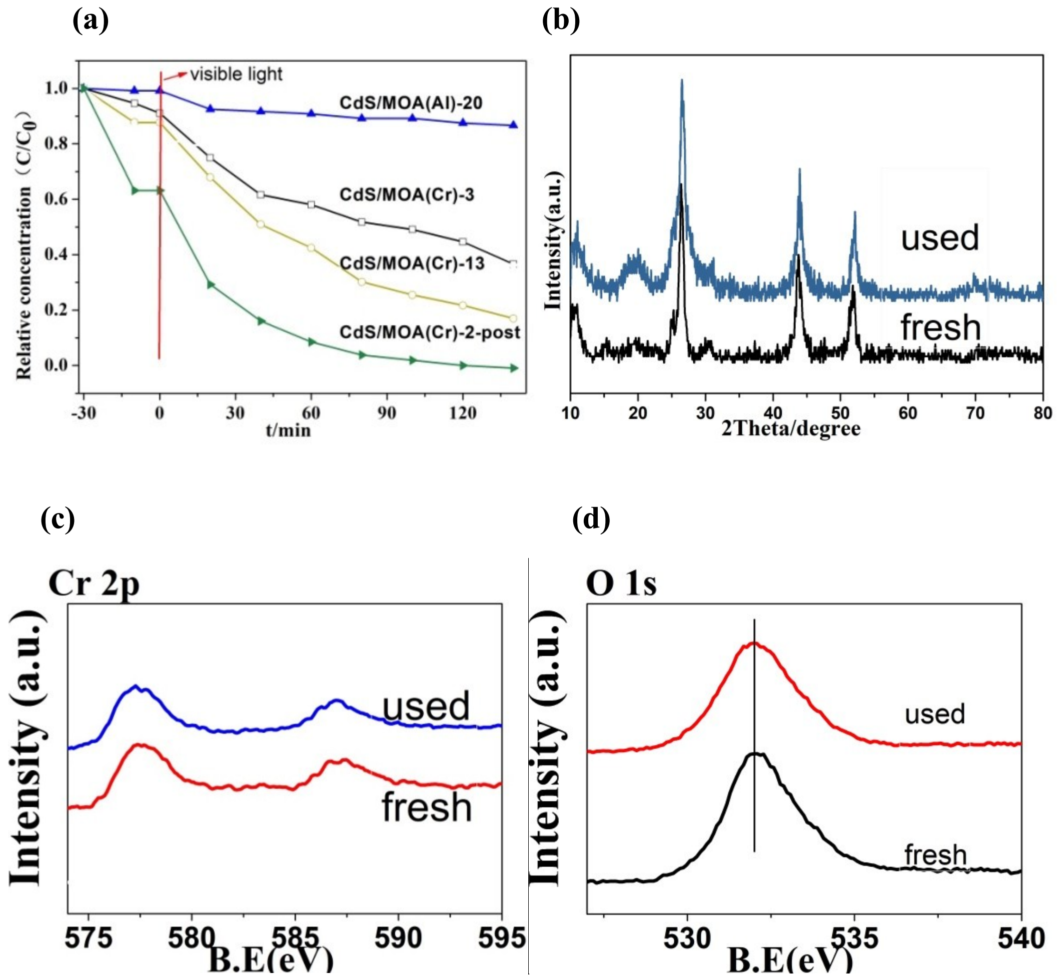

Fig.S5.(a) Photocatalytic activities of photocatalysts for the reduction of Cr(VI) under visible light; (b) XRD patterns of fresh CdS/MOA(Cr)-20 and after used CdS/MOA(Cr)-20; (c) Cr 2p and (d) O 1s spectra of fresh CdS/MOA(Cr)-20 and after used CdS/MOA(Cr)-20.

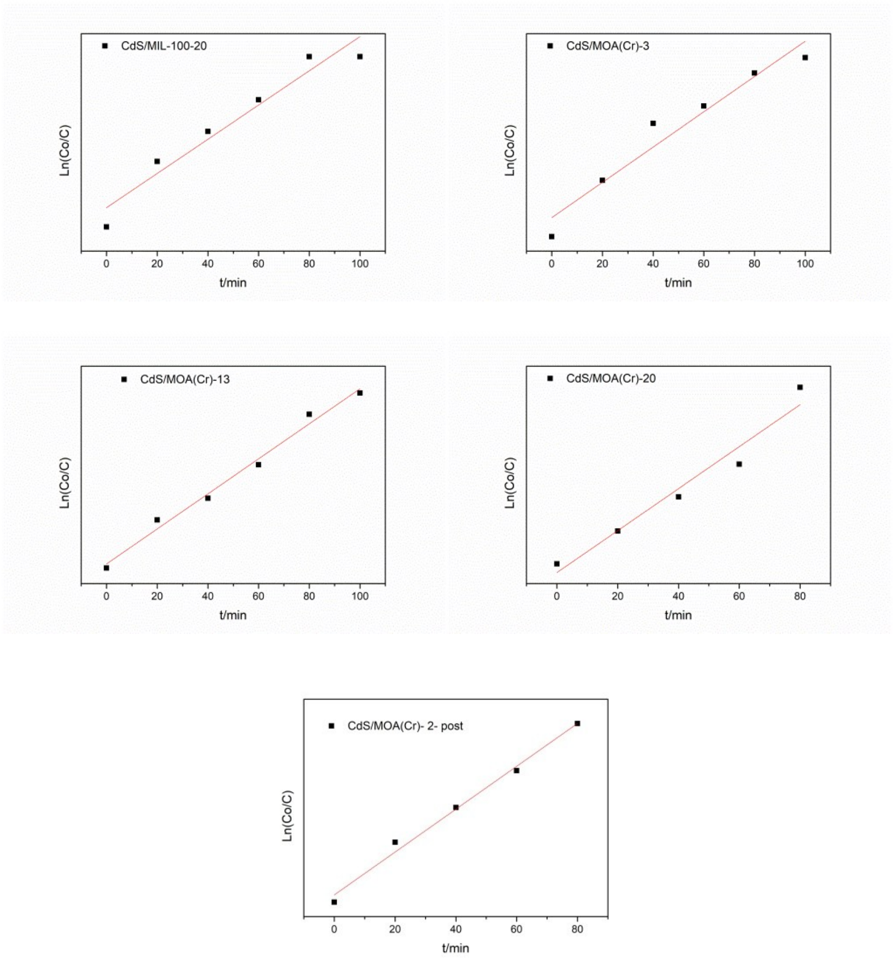

Fig.S6. The curves of In (C_0/C) vs. irradiation time(t)

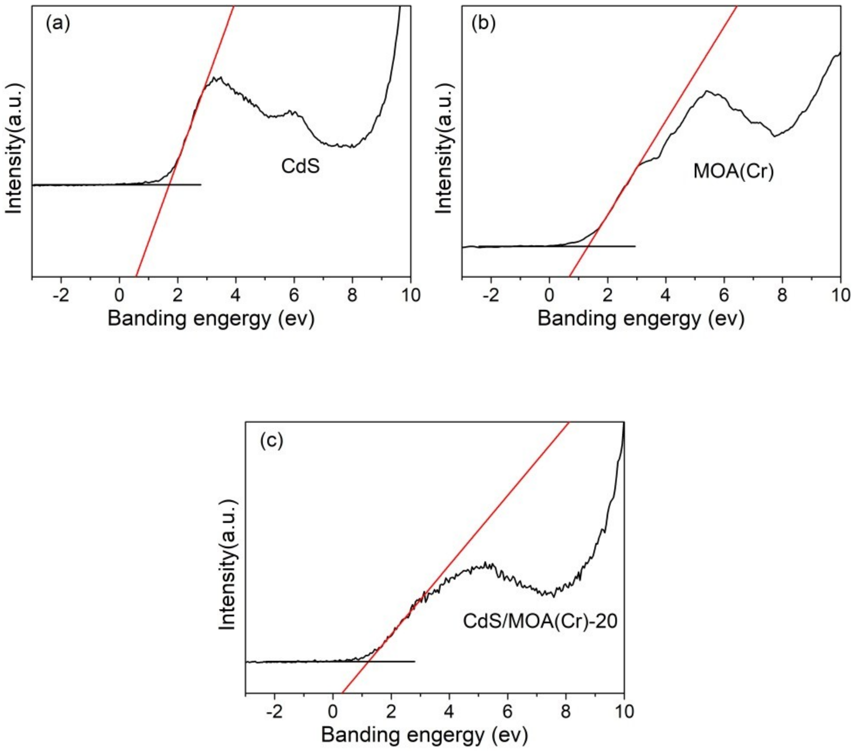

Fig.S7. Valence band XPS spectra of (a) CdS, (b) MOA(Cr) and (c) CdS/MOA(Cr)-20.

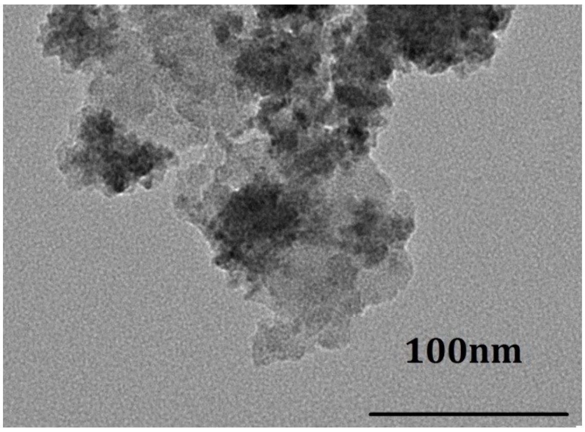

Fig.S8. TEM image of CdS/MOA(Cr)-2-post

[1] G. Ferey, C. Serre, C. Mellot-Draznieks, F. Millange, S. Surble, J. Dutour, I. Margiolaki, Angew Chem Int Ed Engl 2004, 43, 6296.

[2] J. He, H. Yang, Y. Chen, Z. Yan, Y. Zeng, Z. Luo, W. Gao, J. Wang, Water, Air, & Soil Pollution 2015, 226.
